# Modification of Surface Properties of Non-Woven Polypropylene Fabrics by Gaseous Plasma Treatment—Review and Challenges

**DOI:** 10.3390/polym18151886

**Published:** 2026-07-31

**Authors:** Gregor Primc

**Affiliations:** Department of Surface Engineering, Jozef Stefan Institute, Jamova Cesta 39, 1000 Ljubljana, Slovenia; gregor.primc@ijs.si

**Keywords:** non-woven, polypropylene, gaseous plasma, modification, virucidal, XPS

## Abstract

The scientific literature on plasma methods for modifying the surface properties of non-woven polypropylene (NWPP) fabrics is reviewed. The scientific background of the observations reported by different teams is explained, and the technological limits are highlighted. Plasma treatment usually modifies the surface layer, which is beneficial for some applications, such as grafting functional coatings onto the fibers in the surface film of NWPP fabrics. The water contact angle of NWPP fabrics treated by plasmas sustained by the classical dielectric barrier discharges at atmospheric pressure and low-pressure discharges in the range of about 10 to a few 100 Pa rarely drops below 90°, which is explained by the inability to modify fibers deep in the fabrics due to the limited penetration depth of such plasmas. The super-hydrophilic finish can be achieved either by using nanosecond-pulsed atmospheric-pressure discharges or by weakly ionized plasma with a relatively high electron temperature, sustained at a pressure of a few Pa or below. Such plasmas modify the fibers deep in the fabric, which is particularly useful for applications in respiratory masks where the fibers should be coated with ultra-thin films of virucidal substance. The energy efficiency of the latter plasmas is better because practically no gas-phase loss of reactive species occurs, and so is their scalability, making them the most suitable for modifying NWPP fabrics at an industrial scale. While most authors reported only increased wettability with increasing treatment intensity, over-treatment has been reported and is attributed to thermal effects. The range of optimal intensity has yet to be systematically quantified.

## 1. Introduction

Polypropylene (PP) is a lightweight, durable thermoplastic. Due to its chemical inertness, adequate mechanical properties, and low production cost, it is extensively used in packaging, automotive parts, medical devices, and textiles [1]. The latter are often non-woven because these textiles are the least expensive to manufacture and are widely used in single-use products. The products include surgical masks, medical dressings, disposable diapers, wipes, and sanitary napkins [2]. In the automotive industry, non-woven polypropylene (NWPP) is used for trunk liners, seat upholstery, and sound insulation. NWPP is often used in high-efficiency air and water filters [3,4]. Other applications include roofing materials, house wraps, protective crop covers, and biodegradable mulch mats. The mechanical properties of NWPP are adequate for such applications, and so are the chemical properties, as long as the material properties are not intended to be further enhanced by application of various coatings. In the latter case, however, the chemical inertness poses an obstacle, as many materials do not adhere well to PP [1]. In such cases, the surface properties of PP should be modified before applying further treatments.

An important surface property of solid materials is wettability. Wettability refers to a liquid’s ability to completely cover a product, including gaps, pores, and other morphological features. Wettability is often expressed in terms of hydrophilicity, which literally means “wettability for water”. Other expressions are less common, such as oleophilicity [5] or lyophilicity [6]. Wettability depends on the surface energy of the solid and the surface tension of the liquid. Materials will be wettable for a particular liquid if the surface energy of the solid material is at least comparable to (preferably much larger than) the surface tension of the liquid. The degree of wettability depends on the difference between the surface energy of the solid material and the surface tension of the liquid [7]. If the solid surface energy is much larger than the liquid surface tension, the material becomes super-wettable, meaning that a droplet of the liquid spreads rapidly across a large surface soon after deposition.

Wettability is a macroscopic property of a solid material that depends on its micro- and nanoscopic surface properties. The surface energy is the sum of the dispersive and polar components. The dispersive component arises from the simple fact that surface molecules feel an attractive force from molecules below the surface, but there are none above. The dispersive component represents a non-negative and non-zero contribution to the total surface free energy [8]. The polar component arises from the polarity of the chemical bonds at the surface of the solid material. A good example of a non-polar polymer is Teflon. Polytetrafluoroethylene (Teflon) exhibits the lowest total surface energy of 21 mJ/m^2^ among all polymers [9], and oxygen or nitrogen-containing polymers will exhibit a surface energy of roughly 40–50 mJ/m^2^ [10,11,12,13]. The isotactic PP exhibits a surface energy of 32.8 mJ/m^2^, making this material one of the most hydrophobic among all polymers [14]. The polar component of the PP’s surface energy is immeasurably low (practically 0) because of the absence of polar groups on its surface. The water surface tension is about 73 mJ/m^2^.

Wettability does not depend only on surface composition and structure but also on sub-micrometer-scale roughness [15]. In fact, only the rough surfaces exhibit super-hydrophilic or super-hydrophobic surface properties (for highly polar and non-polar surfaces, respectively). Super-hydrophilicity is explained by capillary forces that draw the liquid into the solid’s surface morphological features, causing the droplet to spread over a large area [16].

The surface energy of smooth solid materials is estimated using one of the simplest experimental techniques: depositing a droplet of a liquid with well-determined surface tension and measuring its contact angle. At least two liquids with different surface tensions should be deposited on the surface to calculate the surface energy. While the technique is simple, interpreting the measured values is not trivial and remains a subject of scientific interest [17]. Also, surface contaminants will change the wettability.

**Figure 1:**
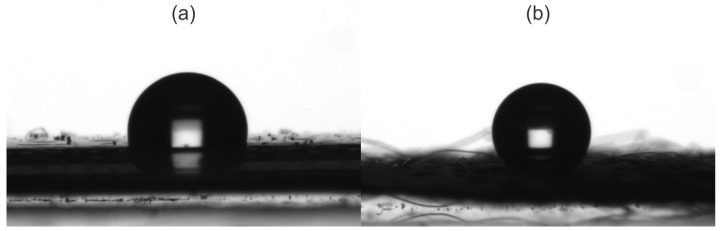
A water droplet on a smooth foil (LS377860 V C S) of thickness 0.27 mm provided by Goodfellow Ltd. (Pittsburgh, PA, USA) (**a**) and NWPP fabrics of thickness 0.2 mm provided by Omega Air d.o.o. (Ljubljana, Slovenia) (**b**). The water droplet with a volume of 2 µL was deposited at ambient conditions (22 °C, 55% relative humidity) using a professional device, DSA 100 E (Kruess, Hamburg, Germany). The photos were taken 5 s after the water droplet was deposited.

Smooth PP without surface impurities will exhibit the water contact angle (WCA) of about 100° [18]. A photo of a water droplet on a smooth PP foil with the WCA of 105° is shown in Figure 1a. The same material, in non-woven form, will exhibit a larger WCA due to its rich micrometer-scale morphology. A photo of a water droplet on a commercial PP textile used for low-cost respiratory masks is shown in Figure 1b. The WCA in Figure 1b is 135°. Clearly, wettability does not depend solely on surface energy (it is pure PP in both cases), but also on morphology.

**Figure 2:**
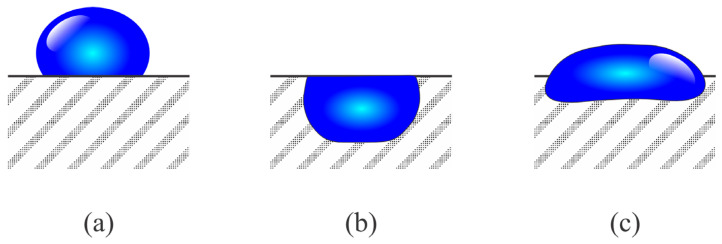
Illustration of the water droplet deposited on untreated (**a**), well-hydrophilized (**b**), and surface-only-hydrophilized (**c**) NWPP fabric.

Taking the above considerations into account, NWPP fabrics will absorb a water droplet if the textile’s fibers are well-hydrophilized. If they are not, a water droplet will remain on the fabric surface and slowly evaporate long after it was deposited. If only the fibers on the surface are hydrophilized, and the rest of the fabric remains hydrophobic, the water droplet will spread on the surface and be absorbed only in the surface film of the fabric, i.e., the film containing hydrophilic fibers. This hypothesized behavior is illustrated in Figure 2. Some examples of the three hypothetical behaviors will be presented below, referring to the available literature.

## 2. Respiratory Masks

Respiratory masks often employ filters made from NWPP. Ideal protective masks should inactivate viruses, be breathable, be non-toxic, be reusable, and be inexpensive. The first two requirements, i.e., inactivation and breathability, are contradictory because masks with a very high filtering efficiency have low breathability. Viruses are sub-microscopic, so the mask filtering layer should be dense enough to capture them and bind them to the fibers over a period of virus infectivity, which is several days for many respiratory viruses at low temperature [19].

Surgical masks are inexpensive and widely used nowadays to protect against airborne particles, including dust, pollen, respiratory virus droplets, bacteria, and fungal spores. A general problem with all standard masks is that they only capture infectious pathogens and do not inactivate them. During the COVID-19 pandemic, wearing masks was compulsory in most countries but did not ensure optimal protection, possibly because the microorganisms remained infectious for hours, if not days, after being captured on the NWPP filter.

The effective filter layer of inexpensive masks is usually made of melt-blown NWPP. This thermoplastic material is melted and spun into filaments, which are randomly joined into sheets during the melt-blowing process, resulting in very good filtering properties. Melt-blown microfibers generally have diameters of about 1 μm [20], enabling them to capture water droplets (as illustrated in Figure 2a) that may contain infectious viruses effectively. The viruses remain on the filter surface and may detach over time with prolonged mask use. An ideal solution is to inactivate any microorganisms upon attachment to the filter, which can be achieved by coating the fibers in NWPP with a germicidal coating. Various approaches to creating such materials and their interactions with microbes have been reviewed recently [21,22].

Surgical and respiratory masks capture relatively large water droplets on their outermost layer, as shown in Figure 2a. Due to evaporation, water droplets slowly shrink, and the smaller ones remain on the surface if adhesion forces exceed the aerodynamic forces acting on them from air movement. The evaporation time depends strongly on the air humidity and flow [19]. The adhesion force at the outermost layer of standard masks is insufficient, so droplets will likely penetrate this layer and contact the filter layer. The filter layer is much denser than the outermost layer, so it will capture even smaller droplets whose diameters exceed the inter-fibrous distances in the filtering textile. The filter surface gradually saturates with droplets when filtering air containing relatively dense droplets, increasing aerodynamic forces during breathing and causing larger droplets to split; eventually, the smaller droplets can penetrate the filter. When this happens, the mask’s protective properties are lost. To avoid this effect, users are recommended to replace masks frequently and carefully, avoiding contact with the inner or outer layers; the WHO recommends changing a mask every 4 h or when it becomes damp [23]. Mask replacement frequency depends on the concentration of virus-containing water droplets in the breathing air, temperature, and relative humidity. Due to frequent mask replacements, vast amounts of respiratory masks have been disposed of, often in an environmentally irresponsible manner. Such frequent mask replacement will not be necessary if the virus is inactivated before the filter becomes significantly covered with droplets.

Another effect to consider is droplet drying. As long as the air humidity is below 100% (in all practical cases), the water droplets slowly evaporate and eventually reach a size smaller than the inter-fibrous distance in the mask filter. Such droplets cannot be captured unless they are attracted to fibers via electrostatic forces. It is worth noting a particular type of electret filter that uses electrostatic forces to capture and fixate water droplets [24]. The droplets containing the viruses remain on the fiber surface as long as electrostatic forces are stronger than aerodynamic forces, which push the droplets in the direction of air movement. Sooner or later, the droplets become small enough to detach from the fibers and continue their path through the mask towards the wearer. Since respiratory viruses remain active for much longer—even up to a week [25]—compared to the typical evaporation time, it is likely that even the droplets captured on filter fibers eventually penetrate the mask, carrying active viruses. The penetration does not pose a health risk if the virus is inactivated before the water droplets reach their penetration volume.

Numerous authors quickly recognized the inadequacies of commercial respiratory masks for personal use, and several groups worldwide reported methods to improve virus capture and/or inactivation. An excellent review of options was published by Tunon-Molina et al. [26]. Various research groups suggested numerous solutions, and a brief overview is presented.

As early as May 2020, Celina et al. proposed spraying virucidal agents on respiratory masks [27]. Some spray-on alcohol-based disinfectant solutions were gently applied to the outer surfaces of the masks to inactivate viruses. They also probed the thermal stability of the masks and reported that most masks have good material properties to withstand a few cycles (i.e., 30 min disinfection steps) of thermal exposure at 75 °C for a few hours, so heating was recommended. Neither moderate thermal aging nor disinfectant spraying caused measurable changes in filtering efficiency, which is not surprising. Another team proposed spraying hypochlorite on surgical masks [28]. A sodium hypochlorite concentration of about 0.2 g/L effectively inactivated the SARS-CoV-2 surrogate viruses, but the authors did not report any tests in a real environment. Furthermore, using hypochlorite imposes an environmental burden [29] and may pose a hazard to the user through inhalation.

Hewawaduge et al. proposed impregnating mask filters with copper sulfide [30]. The copper ions were slowly released upon absorption of virus-containing droplets and inactivated the viruses in about half an hour. The authors claimed their solution would be a lifesaver during the global pandemic but never reported on the production of such masks. Jung et al. also reported copper as an effective sterilizer [31]. A decade ago, Borkow et al. proposed respiratory masks with copper oxide particles [32]. They achieved excellent results for the human influenza A virus and avian influenza virus under simulated breathing conditions. No infectious human influenza A viral titers were recovered from the copper oxide-containing masks within 30 min. Unfortunately, the authors did not conduct cytotoxicity tests. Namely, copper is known for its cytotoxicity [33]. Other metal oxides may also be appropriate [34].

Paranthaman et al. also reported that N95 respirator masks may effectively filter virus particles but cannot kill or inactivate the virus [35]. They disclosed soaking the filter textile in an organosilane-water-stabilized quaternary ammonium chloride formulation containing a non-ionic surfactant with long alkyl chains. The siloxane molecule forms a non-polar covalent bond between the surfaces of masks and filters. They reported applicability at temperatures up to 50 °C and claimed that coated masks continuously inactivated the virus over 3 days but did not publish any inactivation curves. A similar but simpler method using quaternary ammonium salts was recommended by Selwyn and colleagues [36]. The adhesion might not be adequate due to a mismatch in surface energies.

Ludwig-Begall et al. compared germicidal ultraviolet light, vaporized hydrogen peroxide, and dry heat to decontaminate face masks and filtering respirators contaminated with a SARS-CoV-2 surrogate virus [37]. The masks were heated to over 100 °C for an hour, or exposed to high doses of ultraviolet-C (UV-C) radiation, or exposed to H_2_O_2_ vapors. Not surprisingly, all three techniques were found to be appropriate, as the concentration of active viruses dropped by three orders of magnitude. Unfortunately, the methods are not feasible for mass application.

Shan et al. proposed reusable self-sterilization masks based on electrothermal graphene filters [38]. A conductive cloth tape was patterned and adhered to the surface of a filter layer made of melt-blown non-woven fabric, which served as interdigital electrodes. A graphene layer with premier electric and thermal conductivity was deposited onto the fabrics. They mixed a resin with reduced graphene oxide and deposited the suspension onto the conductive cloth. The suspension filled the gaps between the fibers and, after drying, formed a conductive film that was resistively heated to about 80 °C with a 3 V battery. The elevated temperature was advantageous for inactivating the microbes. However, the masks are not particularly suitable for wide-scale manufacturing.

Faucher et al. proposed a reverse-flow reactor concept for the thermal inactivation of SARS-CoV-2 [39]. They introduced a new virucidal face mask concept driven by oscillatory airflow from human breathing. A 3-log reduction in viral load, with minimal impedance within the mask mesh, was observed after heating to 90 °C. Heating masks to such high temperatures is not practical but effective for virus inactivation, as reported by Ludwig-Begall [37].

Lee et al. employed carbon nanotubes as an antiviral agent [40]. They found that the tightly knit carbon nanotube network has pores smaller than the average coronavirus. Surprisingly, the breathability of the innovative filters was equal to that of the conventional PP filter.

Givirovskaia et al. proposed photocatalytic masks [41]. They impregnated filters with zeolites and exposed them to ultraviolet radiation. They found rapid inactivation of selected microbes and attributed the results to photocatalytic effects, i.e., the formation of species with high oxidation potential, rather than to direct interaction with UV photons. It is not practical to irradiate masks with UV radiation in any application.

Regarding research on PP, antimicrobial PP has been prepared through pre-treatment with tannic acid [42], rose Bengal [22], polymeric coatings [22,42,43], embedding nanoparticles [44,45], antimicrobial drugs [44], metals [22,46], etc. Besides research approaches to develop new antimicrobial PP, commercial companies already have their own antiviral masks on the market, containing metal oxides (e.g., Cupron—Cu_x_O, Nexera—AgCu, Sonovia—ZnO, etc.) and other compounds (e.g., GSK—citric acid) with known biocidal action, demonstrating the maturity and readiness of the market to uptake such technologies [47].

The reviewed articles in this section highlight only some of the most exciting methods for improving the properties of respiratory masks. Most authors reported attaching different materials to PP fibers to enhance their germicidal properties. The coatings are often deposited using wet treatments (either dipping in a solution or spraying). The adhesion of such coatings was rarely reported in the reviewed articles, yet it should be inadequate given polypropylene’s chemical inertness and low surface energy. The main reason, as shown in Figure 1b, is the inability of a liquid droplet to penetrate the textile due to its highly hydrophobic nature.

## 3. Hydrophilization of Non-Woven Polypropylene by Gaseous Plasma Treatment

The adhesion of a coating deposited on NWPP textiles by dipping in or spraying with polar liquid suspensions and emulsions will improve as the substrate wettability increases. The increase in wettability is only possible by increasing the surface energy’s polar component, which is achieved by the formation of polar functional groups on the PP surface. Several methods have been used, such as chemical treatment and irradiation with energetic particles (usually UV photons), but treatment with non-equilibrium gaseous plasma has become increasingly popular in recent decades and is widely used today. Plasma treatment is often regarded as fast, environmentally benign, and inexpensive, especially if the equipment cost (plasma reactor with accessories) is marginal compared to other production- or modification-related costs. Plasma could be sustained by various gaseous discharges. They operate across a range of voltages and frequencies, from direct current (DC) to microwave (MW). Low-pressure plasmas are usually sustained either by DC or high-frequency discharges in the MHz to GHz range, because they assure rather temporary uniform plasmas. Atmospheric pressure plasmas (including very popular plasma jets—APPJ), however, are often sustained in the range of kHz. Such plasmas are not uniform but rather appear stochastically.

The treatment of smooth PP objects (usually foils) using non-equilibrium plasma has been a subject of scientific interest for decades. A recent review indicates a broad range of discharge parameters used by various teams to sustain plasmas under conditions adequate for the hydrophilization of smooth PP samples [48]. The authors used the sessile drop method to determine the macroscopic properties of PP treated under various conditions (i.e., the wettability and surface energy) and advanced surface characterization techniques to identify modifications at the atomic and larger scales. The surface composition and structure were usually probed by X-ray photoelectron spectroscopy (XPS), secondary ion mass spectrometry (SIMS), and Fourier transform infrared spectroscopy (FTIR), with the latter technique not providing the surface sensitivity of XPS or SIMS. The morphological changes in smooth PP induced by plasma treatment were typically characterized using atomic force microscopy (AFM) and scanning electron microscopy (SEM). The combination of these techniques, together with appropriate plasma characterization, provided a clear insight into the hydrophilization kinetics. The results reported by different teams are not always in agreement, but the general message is that numerous plasmas can modify surfaces without affecting the bulk polymer properties. Plasma treatments form polar groups on the surface of smooth PP, and prolonged treatment leads to etching. The intermediate step, sometimes overlooked, is the formation of low-molecular-weight fragments (LMWF), which can be removed by a brief wash. Surface functionalization will improve coating adhesion, but the fragments are likely to worsen it, so overtreatment of smooth PP objects is not recommended [49].

The methods used to study the hydrophilization of smooth PP were rarely used by research teams studying plasma methods to increase the wettability of very rough materials, such as NWPP. For example, Ren et al. [50] elaborated on fabric hydrophilization but measured WCA on foils. The limitation of the sessile drop method is well illustrated in Figure 1: a water droplet on a smooth PP exhibits a contact angle that differs significantly from that on NWPP fabrics (the same material but with a different morphology). The water contact angle depends on roughness, so it does not provide sufficient information to calculate surface energy. A droplet of non-polar liquid is likely to be absorbed into the non-woven fabric soon after deposition, so the contact angle cannot be determined. The same applies to the water droplet on well-activated fabric, as illustrated in Figure 2b. The contact angle of the water droplet on surface-only hydrophilized PP fabric is difficult to interpret because of the effect illustrated in Figure 2c.

Surface-sensitive techniques on the atomic or molecular scale (XPS, SIMS) also fail to yield adequate results due to the complex morphology. Surface charging is effectively compensated for smooth samples using electron guns for surface neutralization, but it is problematic for very porous materials, as explained in the classical paper by Mozetic [51]. Not surprisingly, only a few research groups that used plasma treatment of NWPP to improve coating adhesion reported the evolution of surface wettability or surface functional groups as a function of the treatment parameters.

### 3.1. Literature Without Reports on NWPP Wettability, Structure, or Composition

Plasma treatment of NWPP has been reported to improve the adhesion of various coatings. One of the first reports on the hydrophilization of NWPP was provided by De Geyter et al. [52]. The team studied the penetration of gaseous plasma inside textiles. This topic is very important, as illustrated in Figure 2. Reactive plasma species are generated in the discharge and often do not penetrate gaps, pores, cracks, and similar morphological features. The reasons are explained in detail in [53]. De Geyter et al. [52] used a dielectric barrier discharge (DBD) to sustain plasma in synthetic air (nitrogen and oxygen, without other gases) at pressures between 300 and 7000 Pa. The NWPP textiles, three layers thick, were placed on a grounded electrode, which was covered with a thin glass plate that served as a dielectric barrier to maintain the discharge in filament mode at elevated pressures. At low pressure, the uppermost layer was well activated, but at higher pressures, the lowermost layer was better activated. The reason was the proximity of the lower layer to the electrode, which led to streamer formation there. Low-pressure plasma, on the other hand, is characterized by a rather uniform plasma that cannot penetrate pores [54].

**Figure 3:**
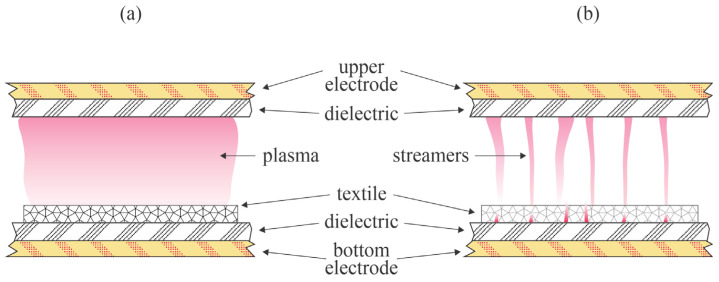
The effect of fabric treatment with plasma sustained by DBD at low (**a**) and high (**b**) pressures.

The effect studied by De Geyter et al. [52] is illustrated in Figure 3. At low pressure, a semi-continuous plasma forms in the volume between the electrodes, as shown in Figure 3a. There are hardly any short-duration and high-power streamers, so the density of charged particles is too low for plasma to penetrate deep into the pores between the textile fibers. Namely, the plasma could be sustained in the pores only if the density of charged particles is large enough to make the Debye length shorter than the distance between the fibers. A typical inter-fiber distance in NWPP used for masks is of the order of micrometers. The Debye length decreases as the square root of the electron density and is 0.658 µm at the electron density of 1.1 × 10^20^ m^−3^ and temperature of 10,000 K. Only very powerful discharges can sustain such a high electron density in a continuous discharge; the application of plasmas sustained in a continuous mode is not realistic for the treatment of porous polymers. Namely, the energy flux at the electron density of 10^20^ m^−3^ will melt the surface fibers in a fraction of a second. The energy flux is the production of a particle flux and the energy brought to the surface by a particle. If the particles are positive ions and the ion density equals the electron density (true for all plasmas except those sustained in highly electronegative gases), an ion will carry the potential and kinetic energy. The potential energy equals the ionization energy (typically 10–20 eV for most gases), and the kinetic energy corresponds to the potential drop in the sheath adjacent to the surface (which depends on the electron temperature and ion mass, typically 10–20 V). In the collisionless heat approximation, the kinetic energy is spent on heating the sample. The energy carried by a positive ion impinging on the sample is thus roughly 30 eV. The flux of ions on the sample surface is a product of density and Bohm velocity, so the power dissipated on the sample surface is between 10^5^ and 10^6^ W/m^2^ if the electron density is 10^20^ m^−3^.

At high pressure, however, plasma cannot be sustained in a continuous mode due to the loss of charged particles in the gas phase. Instead of continuous plasma, the DBD at high pressure causes numerous streamers, which create very short-lived plasma after the ionization wavefront. The streamers originate from the dielectric surface backed by both the upper and the lower electrode, as illustrated in Figure 3b. As a streamer propagates through the gas, its energy decreases due to energy loss from ionization, dissociation, and excitation of gaseous molecules. The density of charged particles in plasma formed after the ionization wavefront decreases as they move away from the electrode. As a consequence, the streamers can penetrate the textile closer to the electrode, but not the textile placed away from the electrode. The net effect is that the textile on the bottom electrode is hydrophilized more efficiently than the textile closer to the upper electrode. The pioneering work reported by De Geyter et al. [52] should be taken into account in any attempt to interpret the hydrophilization of non-woven textiles via plasma treatments.

#### 3.1.1. Treatment with Low-Pressure Plasmas

Blanes et al. [55] used a low-pressure (42 Pa) plasma sustained in a mixture of methane and oxygen by a radiofrequency (RF) capacitively coupled discharge at 150 W for 5 min. The plasma treatment was performed to achieve a surface finish on NWPP to ensure good attachment of nanofiber webs synthesized by electrospinning. The plasma treatment improved surface conductivity, but it was insufficient for good web deposition. The WCA on untreated samples was about 110°, and it dropped to 85° after the plasma treatment. An RF discharge produces a continuous plasma at 42 Pa, so the illustration in Figure 3a can be adopted. The surface finish obtained by Blanes et al. [55] resembles the one illustrated in Figure 2c.

Song et al. [56] treated NWPP membranes with oxygen plasma at 15 Pa (other parameters were not disclosed). The plasma-treated membranes were immersed in a sulfonic acid-terminated monomer solution, degassed at atmospheric pressure under argon flow, and graft polymerization was carried out under a high-intensity UV lamp. The oxygen plasma pre-treatment was found to be adequate for the appropriate adhesion of the grafted coating. A similar plasma pre-treatment was also performed for the graft polymerization of a zwitterionic polymer onto NWPP membranes [57]. Based on Figure 2b, the plasma-induced modification was limited to the fabric’s upper layer, which was sufficient for coating the fabric with the desired chemicals.

Li et al. [58] used oxygen plasma pre-treatment to initiate the graft polymerization of acrylic acid on the surface of NWPP fabrics. The base pressure in the plasma reactor was 4 Pa, and oxygen was introduced to raise the pressure to 15 Pa. The discharge power was 30 W, and the treatment time was 3 min. The treatment conditions were similar to those illustrated in Figure 3a, enabling the modifications as illustrated in Figure 2c. The coating was found to be beneficial for heparin immobilization, which improved hemolysis rates and blood-platelet adhesion, and could potentially be applied to blood-plasma purification and separation. In a separate paper, the same team managed to graft another hemocompatible layer onto NWPP without plasma pre-treatment [59]. UV-stimulated graft polymerization of acrylic acid on the surface of oxygen-plasma-pre-treated NWPP fabrics was also used by Zhang et al. [60] to immobilize bovine serum albumin and thus achieve a hemocompatible polymer surface.

Low-pressure oxygen plasma was used to treat NWPP samples before hematite powder deposition by Tomaszewska et al. [61]. Plasma was sustained by a capacitively coupled RF discharge at a pressure of 5 Pa and a discharge power of 50 W. The configuration was similar to that illustrated in Figure 3a except that the dielectric plates were omitted. The plasma-treated samples were placed in a beaker containing the hematite suspension to allow the powder to adhere. To improve the binding between hematite nanoparticles and the PP matrix, the dry filter was heated to 145 °C (near the PP melting point) for 5 s. SEM confirmed excellent coverage of the polymer fibers with the hematite particles.

Yet another report on the application of low-pressure plasma as an essential step for adequate bonding on nanoparticles was reported by Voznesensky et al. [62]. Plasma was sustained in the air at a pressure of around 20 Pa and RF power up to 2 kW, so the illustration in Figure 3a applies (except the dielectric barrier). Plasma-activated NWPP substrates were impregnated with silver nanoparticles from a colloidal solution, dried, and reinstalled in the plasma reactor. The second plasma treatment was performed in a mixture of argon and propane-butane to deposit a thin film by plasma polymerization. The first plasma treatment improved the sorption properties of PP fibrous materials, ensuring a uniform distribution of nanoparticles with antibacterial activity across the entire sample area. Repeated RF plasma treatment can be used to anchor nanoparticles to the surfaces of polymeric materials.

**Figure 4:**
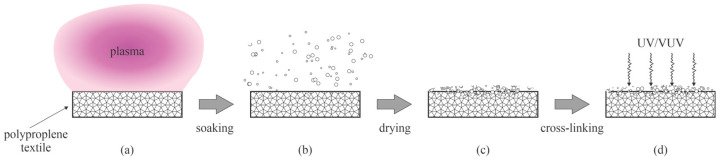
A suitable method for grafting the surface of the PP fabric with a desired coating. (**a**) Fabric is exposed to low-pressure plasma to make the material wettable; (**b**) a suitable liquid wets the fabric surface; (**c**) the fabric is dried so that the desired chemicals remain on the surface; (**d**) post-treatment with UV/VUV enables cross-linkage.

While the previously cited articles disclose deposition of different coatings, the effect of treatment with low-pressure plasma is similar: it enabled hydrophilization of the surface fibers (Figure 2c), which was sufficient to ensure spreading of the water solutions onto the fabric surface and thus the formation of the appropriate coating. Further processing with UV photons enabled polymerization and/or cross-linkage of the deposited films. The procedure is illustrated in Figure 4. Various authors used different methods; therefore, Table 1 indicates which gas, pressure and treatment time were used, as well as the type of reactor, electrical parameters and the PP sample type.

**Figure 5:**
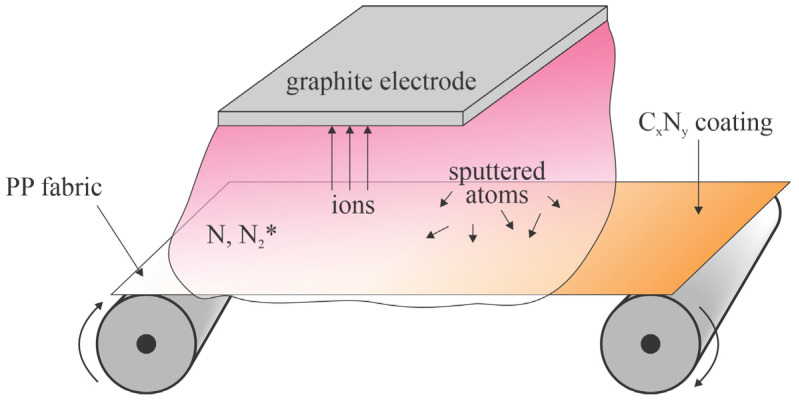
Illustration of the experimental setup for increasing the dispersive component of the NWPP’s surface energy by deposition of a carbon nitride film.

A powerful low-pressure plasma reactor was also used by Sohn et al. [63]. NWPP fabrics were treated in a reactor powered by a pulsed DC discharge in the magnetron mode at an average power of 450 W in continuous mode (roll-to-roll). Plasma was sustained in a mixture of argon and nitrogen at a pressure as low as 0.23 Pa. The energetic ions caused sputtering of the graphite cathode and the growth of uniform carbon nitride films. The roll speed of 100 mm/minute enabled the effective deposition time of 20 s. Sohn et al. [63] measured wettability using two liquids (water and diiodomethane) and determined the surface energy before and after plasma treatment. The diiodomethane droplet contact angle was immeasurably low after plasma treatment, but the water droplet shape did not change much (the WCA of untreated PP fabrics was about 130°). The contact angles enabled the determination of both the polar and dispersive surface energies. The polar component was marginal before and after plasma treatment, but the dispersive component increased from 23 to 60 mJ/m^2^. The schematic of the experimental configuration employed by Sohn et al. [63] and the effects of different reactants are illustrated in Figure 5.

#### 3.1.2. Treatment with Atmospheric Pressure Plasmas

According to Figure 3, atmospheric-pressure plasmas may penetrate deeper into non-woven textiles, so appropriate parameters may ensure the conditions illustrated in Figure 2b. Plasma at atmospheric pressure is dominated by gas-phase collisions because of the short mean-free path (roughly 10 micrometers) and the short time between successive collisions (roughly 10 ns). Furthermore, the three particles are likely to collide at atmospheric pressure. As a result, the particles with a large potential energy are likely to de-excite (lose their potential energy) in the gas phase. For example, the O atoms are lost by the super-elastic collisions O + O + O_2_ → O_2_ + O_2_. The potential energy (due to dissociation) before the collision is transformed into the kinetic energy of the molecules after the collision. The net effect of gas-phase collisions is heating of the gas rather than chemical interactions between plasma species (radicals, ions) and the object exposed to the plasma. Two- or multi-atom molecules are more likely to dissociate or be excited than to be ionized under plasma conditions because of the low electron temperature and the high ionization threshold. The loss is significantly suppressed if the plasma is sustained in a noble gas. Namely, in noble gases, the available discharge power is predominantly spent on ionization or the excitation of metastables, so plasma could be sustained at a reasonable power in much larger volumes than when using gases of multi-atom molecules. The noble gases do not interact chemically with the solid material, so a small admixture of a reactive gas is used to trigger surface chemical reactions.

Ye et al. [64] used plasma sustained in a mixture of 99 vol.% argon and 1 vol.% air at atmospheric pressure to treat the NWPP mesh. Plasma was sustained by a standard DBD, so the simple illustration shown in Figure 3b applies. Plasma-treated samples were immersed in benzophenone and irradiated with UV photons. Afterward, glucose ligands were bound to the surface. Such a coating enabled a highly hydrophilic surface, rapid adsorption kinetics, and a high protein-binding capacity, which was found useful for biomedical applications. The procedure was similar to that in Figure 4 except that the liquid might penetrate throughout the fabric.

Yang et al. [65] used two types of atmospheric-pressure plasmas sustained in air to render NWPP substrates hydrophilic: a standard DBD (as illustrated in Figure 3b) and a nanosecond-pulsed discharge. The latter is characterized by numerous very short current pulses that propagate between the electrodes covered with a dielectric. The WCA of untreated PP was 145°, and it dropped to 110° after receiving an energy density (energy per geometric surface area) of 0.1 J/cm^2^ using nanosecond-pulsed discharge. This is about 20 times lower energy density than needed to achieve the same wettability with the standard DBD powered by a sinusoidal AC voltage at 10 kHz. This is one of the few reports on surface damage to non-woven fabric induced by plasma generated by a dielectric barrier discharge. As illustrated in Figure 3b, the streamers hitting the fabric surface are likely to dissipate energy at the surface, thereby imposing a thermal load that may melt the fibers at the impact spot. The evolution of the WCA with respect to the energy density was shown, but the authors did not report the discharge power or treatment time, so the results are not comparable to those in the next subsection (3.2) of this article.

**Figure 6:**
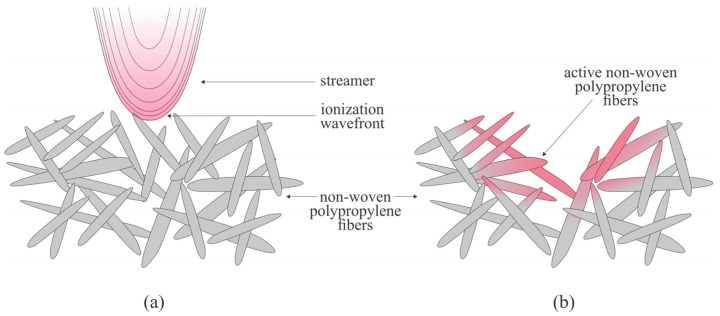
The effect of a gas streamer from a standard DBD upon impact on NWPP fabric: (**a**) before, and (**b**) after impact.

The minimum WCA of 60° was observed at an energy density of 60 J/cm^2^ when using the standard DBD, but visible damage was observed. The effect of plasma treatment of NWPP when using the standard DBD is illustrated in Figure 6. The non-woven fabric consists of randomly oriented PP fibers with little or no thermal contact between them. When the ionization wavefront touches the surface, the charge is distributed onto the fibers, and the electric field becomes too weak to sustain plasma in the space between the neighboring fibers. The streamer energy is thus dissipated on the surface fibers, which are heated for a short time. The streamer energy (especially when using argon with a small admixture of a reactive gas) is large enough to cause localized melting of the fibers. The SEM micrographs provided by Yang et al. [65] clearly showed the formation of such holes and other morphological features that are absent in untreated textiles. Only the fibers hit by dense plasma just after the ionization wavefront will melt. Around this spot, there will be diffusing plasma species whose density is too low to cause significant heating but high enough to cause surface functionalization of the fibers around the impact spot. The effect is illustrated in Figure 6b: around the hole caused by localized fiber melting, there will be a volume of functionalized fibers. Numerous streamers occur in standard DBDs (as a rule of thumb, 10^5^ s^−1^ at the discharge frequency of 10 kHz), so the final effect of such treatment will be the same as illustrated in Figure 2c.

**Figure 7:**
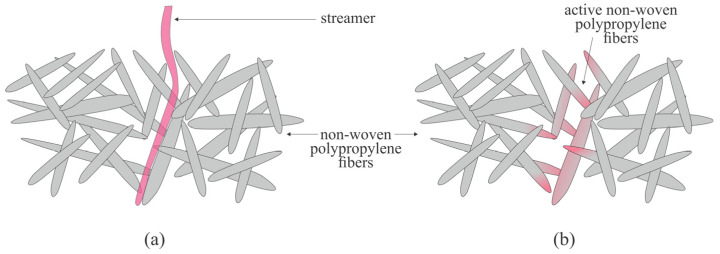
The effect of a gas streamer from a nanosecond-pulsed discharge impacting a NWPP fabric: (**a**) before, and (**b**) after impact.

Yang et al. [65] also used a discharge that produces much more focused streamers. The pulsed DBD produced a super-hydrophilic surface after a dose of 10 J/cm^2^, as illustrated in Figure 2b. The fundamental difference between the results of the two discharges is evident in Figure 6 and Figure 7. Figure 7 shows the effect of a streamer formed in a nanosecond-pulsed discharge. The streamer is much smaller, and the electric field in front of the ionization wavefront is much larger than in the case of standard DBD (Figure 6a). The nanosecond streamer will thus be able to propagate through the textile, so the final effect of a single streamer will be as shown in Figure 7b. Numerous streamers appear in nanosecond-pulsed discharge (at least one per pulse), so the net effect of the treatment will be as shown in Figure 2b—functionalization throughout the fabric and thus an immeasurably low WCA. In fact, Yang et al. [65] reported the super-hydrophilic character of the treated NWPP after receiving the energy dose of about 10 J/cm^2^.

Atmospheric-pressure plasma sustained in ambient air was used by Markovic et al. [66] to treat NWPP fabrics in a semi-continuous mode. The fabrics passed a plasma zone sustained by a corona discharge. The plasma-treated samples were immersed in a colloidal titania nanoparticle solution with photocatalytic activity. The adhesion of nanoparticles to the plasma-treated NWPP was found to be adequate for the photocatalytic degradation of simple organic molecules in water. Streamers caused by the corona discharge are likely to be much more energetic than those by DBD, so the effect similar to that shown in Figure 6b is likely to occur. The fundamental difference between DBD and corona discharges is in the way the electric current is limited: a resistor placed in series limits the current in the corona discharge, and the dielectric plays this role in DBD.

### 3.2. Evolution of the Wettability for Plasma-Treated Non-Woven Polypropylene

**Figure 8:**
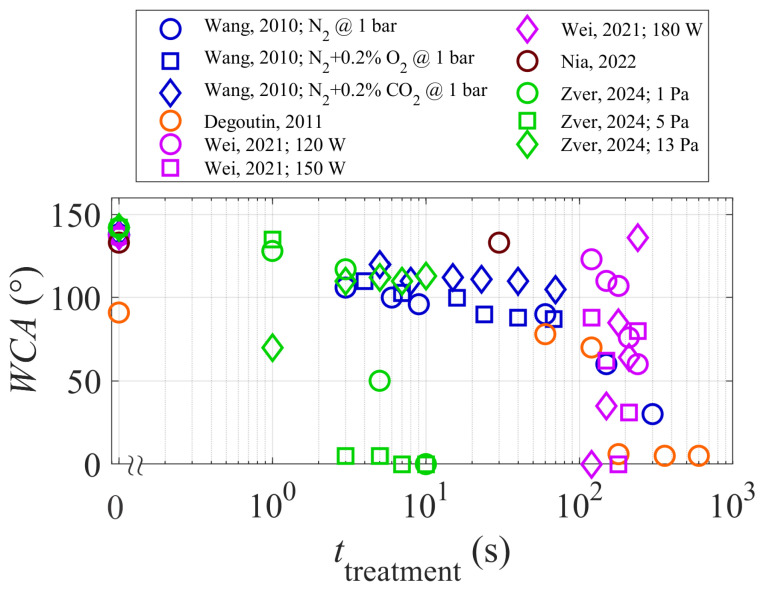
Evolution of water contact angles on NWPP with plasma treatment time. Treatment conditions: Wang et al. 274 and 294 W, 8 kHz, 31 kV [67]; Degoutin et al. 100 W, 13.56 MHz, 15 sccm Ar [73]; Wei et al. 120, 150 and 180 W, air [74]; Nia et al. 100 W, 50 kHz, O_2_ [75]; Zver et al. 10 W, 13.56 MHz, 1 Pa [76].

**Figure 9:**
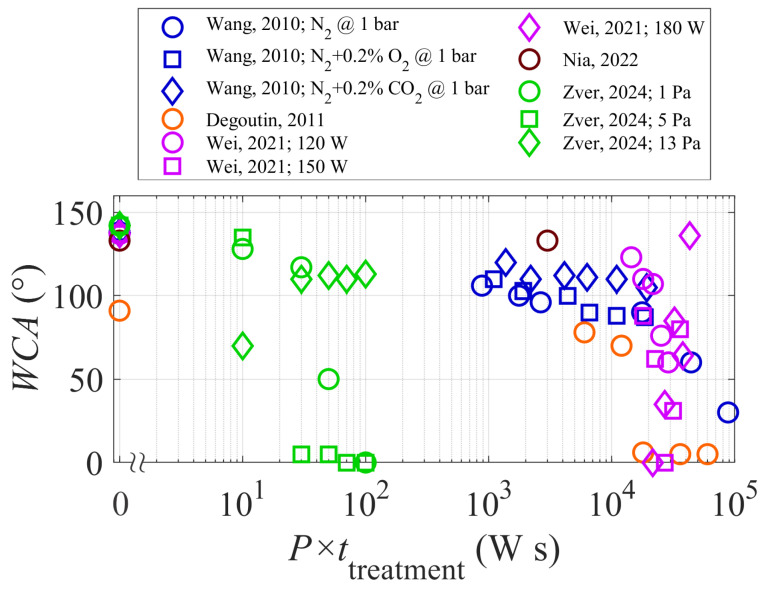
Evolution of water contact angles on NWPP with the product of reported discharge power and the plasma treatment time. Treatment conditions: Wang et al. 274 and 294 W, 8 kHz, 31 kV [67]; Degoutin et al. 100 W, 13.56 MHz, 15 sccm Ar [73]; Wei et al. 120, 150 and 180 W, air [74]; Nia et al. 100 W, 50 kHz, O_2_ [75]; Zver et al. 10 W, 13.56 MHz [76].

Some authors measured the water contact angle on NWPP samples before and after plasma treatments. One of the first reports was provided by Wang et al. [67]. The team used atmospheric-pressure DBD to sustain plasma in either (almost) pure nitrogen or nitrogen with 0.2 vol.% of oxygen or carbon dioxide. The discharge operated at 8 kHz and 31 kV, and the discharge power varied slightly with gas, ranging from 270 to 290 W. The evolution of the water contact angle was reported as a function of the energy per unit area of the treated samples. The energy density was systematically varied from 1 to 100 J/cm^2^, corresponding to treatment times of roughly 3–300 s. The initial WCA was about 138°. Clearly, the samples were similar to the one shown in Figure 1b. Plasma treatments caused a gradual decrease in the WCA, but energy densities of up to about 20 J/cm^2^ (treatment times up to about 1 min) did not reduce the WCA to well below 90°. Hence, the fabrics retained their hydrophobic character, as illustrated in Figure 2c. Mixing nitrogen with 0.2 vol.% of oxygen enabled better results than pure nitrogen. The WCAs reported by Wang et al. [67] are plotted in Figure 8 and Figure 9 with blue symbols. Prolonged treatment (for several minutes) led to a further decrease in the WCA, but super-hydrophilicity was not observed, at least not at the longest treatment time of 5 min (Figure 8). The energy efficiency of the methods reported by Wang et al. [67] is inadequate (Figure 9).

Wang et al. [67] also probed selected samples by XPS. The survey spectra of the untreated PP sample showed 2.7 at.% oxygen. PP is an oxygen-free polymer, so some oxygen on the surface of an untreated sample was attributed to contamination. After 30 s of plasma treatment in nitrogen, the sample surface contained about 64 at.% carbon, 21 at.% oxygen, and 15 at.% nitrogen. Oxygen was found on the PP surface after plasma treatment in oxygen-free gases, even in a carefully designed experiment [68], and is usually attributed to oxidation by gaseous impurities, primarily water vapor. Water molecules dissociate in nitrogen plasma via multiple channels, including electron-impact dissociation and quenching of nitrogen metastables. The OH radicals exhibit a high oxidation potential and interact readily with hydrocarbons by abstracting a H atom, forming a water molecule, and a dangling bond, which is occupied by another OH radical. The net effect is R–C–H + 2OH → R–C–OH + H_2_O. This reaction was found very probable by both theoreticians [69] and experimentalists [70]. An alternative explanation for the high oxygen concentration is post-oxidation of dangling bonds (which may remain on the surface well after the plasma treatment) upon exposure of plasma-treated samples to the ambient conditions. The formation of dangling bonds is attributed to absorption of energetic plasma photons [71] upon exposure to air after the plasma treatment. Nitrogen plasma is a rich source of photons in the 120–175 nm range [72]. The high-resolution C1s peaks reported by Wang et al. [67] for samples treated for 30 s revealed a concentration of C–C or C–H groups on the PP surface of about 65 at.%, while the concentration of the C–O or C–N groups was about 18 at.%. The concentrations of the carbonyl, carboxyl, and carbonate groups were 9 at.%, 5 at.%, and 3 at.%, respectively. Still, such a rich surface functionalization did not translate into excellent wettability, as the WCA was about 90° (Figure 8 and Figure 9).

Ivanova et al. [77] treated NWPP with atmospheric-pressure plasma sustained in air via a coplanar surface barrier discharge. This discharge is similar to DBD, except that the plasma forms only on the surface of the ceramic plate with embedded electrodes. Thus, the plasma streamers do not penetrate the fabrics, helping prevent damage, as reported by Yang et al. [65]. The plasma treatment time ranged from 0 to 10 s. Ivanova et al. [77] found WCA measurements inappropriate (probably due to droplet absorption in the fabrics), so they reported changes in wettability in terms of the strike-through time, i.e., the time required for a polar liquid to penetrate the non-woven fabrics. The results were impressive: while the strike-through time was about 400 s for untreated PP, it dropped to 28, 14, 13, 11, and 9 s for treatment times of 0.5, 1, 3, 5, and 10 s, respectively. The coplanar discharge used by Ivanova et al. [77] is powerful enough to modify fibers deep within the fabric and to achieve the properties illustrated in Figure 2b.

**Figure 10:**
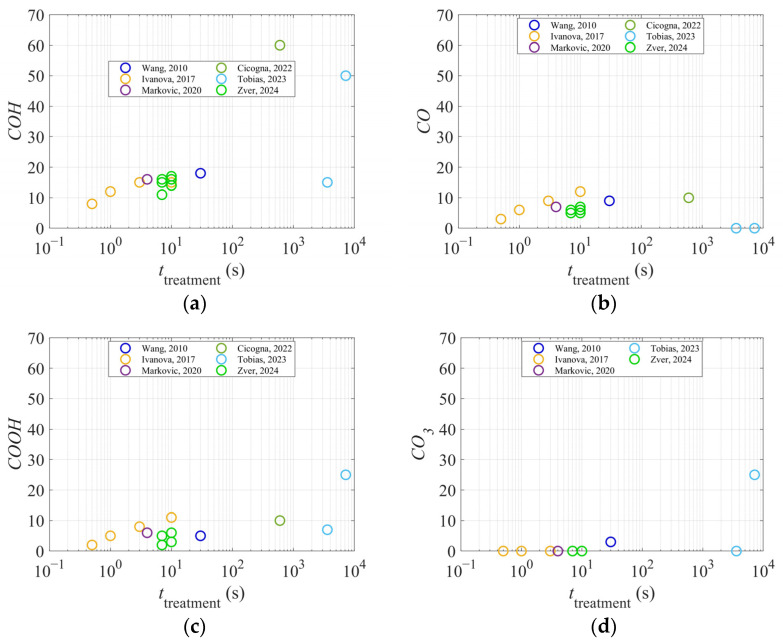
The formation of various functional groups, as deduced from high-resolution C1s XPS spectra, as a function of plasma treatment time. (**a**) C–OH (likely hydroxyl), (**b**) C=O (carbonyl), (**c**) O–C=O (carboxyl or ester), and (**d**) CO_3_ (carbonate). Treatment conditions: Wang et al. 274 and 294 W, 8 kHz, 31 kV, dielectric barrier discharge [67]; Ivanova et al. 300 W, air, coplanar dielectric barrier discharge [77]; Markovic et al. Vetaphone CP-Lab MK II 700 W, air [78]; Cicogna et al. Tucano, Gambetti 150 W, 20 sccm O_2_, 50 Pa [79]; Tobias et al. 40 kHz, air, 70 Pa [80]; Zver et al. 1–13 Pa, inductively coupled plasma [76].

Systematic measurements of the surface functional groups by XPS were performed by Ivanova et al. [77]. The untreated PP samples showed about 2 at.% oxygen, attributed to adsorbed surface impurities, as already reported by Wang et al. [67]. This ratio increased to over 20% even after 0.5 s of treatment. Further treatment led to an increase in oxygen concentration. The O/C ratio reached 0.5 after 10 s of plasma treatment. High-resolution C1s spectra showed the formation of various functional groups. The concentration of different functional groups as a function of plasma treatment time is shown in Figure 10 (yellow circles). The results reported by other authors are added to Figure 10.

Markovic et al. [78] also examined plasma-treated NWPP fabrics by XPS. They used plasma sustained by corona discharge in the air to modify the textiles. The treatment was performed in a continuous mode, i.e., the fabrics were mounted on a roll-to-roll system and passed through the discharge region at 4 m/min. The discharge power was 700 W, and the number of passes through the plasma zone was 30. The width of the plasma zone was not specified, so the treatment time could only be roughly estimated to be about 4 s. Such a treatment time was sufficient to observe the O/C ratio of about 0.25. The concentrations of C–O, C=O, and O–C=O groups after plasma treatment of NWPP were 16, 7, and 6 at.%, respectively.

Wei et al. [74] treated NWPP fabrics with cold plasma sustained in the air. The experimental setup was not disclosed, but the discharge power was varied between 120 and 180 W, and the treatment time between 120 and 240 s. Systematic measurements of the WCA were reported, and the values are plotted in Figure 8 and Figure 9 (purple color). The untreated PP fabrics exhibited a WCA of 137°, similar to that shown in Figure 1b. The low discharge power of 120 W (indicated by purple circles in Figure 8 and Figure 9) resulted in a gradual decrease in the WCA, reaching 60° after 3 min of plasma treatment. The medium discharge power of 150 W (purple squares in Figure 8 and Figure 9) caused a well-pronounced minimum in the curve. Namely, the WCA gradually decreased with treatment time, reached an immeasurably low value at 3 min, and increased steeply afterward. A similar trend was observed at 180 W (purple diamonds in Figure 8 and Figure 9), except that the immeasurably low WCA was observed only for samples treated for one minute. The authors explained the increased WCA after prolonged plasma treatment by the generation of sufficient heat to destroy the fiber-mat structure after a long treatment period. Yet another feasible explanation is the thermal degradation of the polar functional groups. The high polar component of the surface energy is thermodynamically unfavorable, so polar surface functional groups tend to reorient towards the bulk, and the hydrophobic recovery increases steeply with the plasma-hydrophilized polymers’ temperature [81].

**Figure 11:**
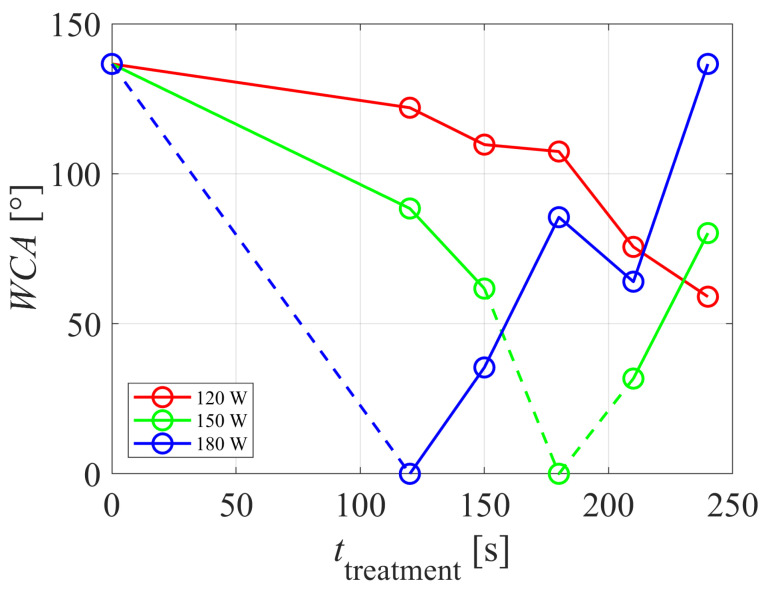
The variation in the WCA reported by Wei et al. [74] with the treatment time. The discharge power is the parameter. The dashed parts of the curves represent values that would need more measured points, but were not reported in sufficient detail by the authors.

Wei et al. [74] achieved various stages, ranging from complete absorption of a water droplet (Figure 2b) to modification of the surface layer only (Figure 2c). The variation in the WCA with discharge power and/or treatment time, from untreated samples (Figure 2a) to moderately treated samples (Figure 2c), was gradual until a critical parameter was reached, which enabled immediate absorption of a water droplet. The effect is illustrated in Figure 11. The measured points in Figure 11 were extracted from the photos of water droplets presented in the article [74]. The solid curves in Figure 11 represent the relation likely to be observed if the measurements were performed between the points (interpolation), and the dashed lines are for eye guidance only. From the results of Wei et al. [74], it is not possible to predict the exact treatment time at which the super-hydrophilic fabric (as in Figure 2b) would appear.

**Figure 12:**
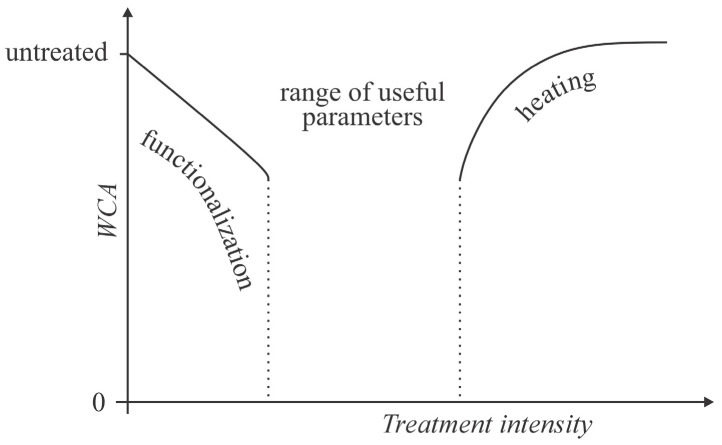
The range of useful treatment intensity for the absorption of polar liquids deep into PP fabrics.

The results reported by Wei et al. [74] clearly show that over-treatment is as bad as under-treatment. Two effects govern the WCA of NWPP: surface functionalization with polar groups and thermal effects. Both increase with treatment time and discharge power but have opposite effects: functionalization decreases WCA, while heating increases it. The net effect is a minimum in WCA, as shown in Figure 12. The solid lines, which represent the effects of functionalization and thermal effects, do not extend to low WCAs due to water droplet absorption, which occurs only over a limited range of treatment intensity. The effect illustrated in Figure 12, with dotted lines, would not be observed for smooth PP and is thus a unique property of the fabrics. Namely, water cannot be absorbed into smooth PP; it only adsorbs and spreads over a surface area that increases monotonically with increasing wettability [48]. The illustration in Figure 12 is quantitative. The quantification poses a major scientific challenge, particularly because the “treatment intensity” does not directly translate into discharge power but rather into the power dissipated at the sample’s surface. The power normalized to the surface area depends on the fluxes of reactive plasma species and the coefficients for the surface exothermic reactions, such as heterogeneous recombination of molecular radicals.

Degoutin et al. [73] pre-treated NWPP with plasma sustained at a low pressure in argon by a capacitively coupled RF discharge at a power of 100 W. The treatment times were up to 10 min. The WCA for the untreated sample was as low as 90°. Such a WCA is typical of moderately hydrophobic smooth polymers and has not been reported by any other authors who have tackled the hydrophilization of NWPP. Plasma treatment caused a gradual decrease in the WCA, which reached 70° after 2 min. Further treatment caused an almost immeasurably low WCA. A sudden drop in WCA is explained by the hydrophilization of the fabrics, as illustrated in Figure 12, where a water droplet is absorbed into the porous structure after reaching the critical treatment intensity. According to the authors, argon plasma treatment produced dangling bonds, which were occupied by oxygen upon exposure to air. An alternative explanation is in situ functionalization due to the residual atmosphere in the low-pressure plasma reactor. The water molecules, which usually represent the residual atmosphere in hermetically tight vacuum systems, are likely to dissociate under plasma conditions, so the effect could be the same as in the experiments by Wang et al. [67]. An almost immeasurably low WCA was measured at any plasma-treatment time between 3 and 10 min, so the hydrophobic recovery (Figure 11 and Figure 12) reported by Wei et al. [74] was not observed. The intensity of surface exothermic reactions during argon-plasma treatment of polymers is much lower than during air-plasma treatment, so the polymers are not heated enough to observe the hydrophobic recovery as illustrated in Figure 12. The plasma-treated samples were grafted with acrylic acid and exposed again to argon plasma to cross-link the deposited film by VUV radiation arising from excited Ar atoms and/or Ar_2_* excimers, as illustrated in Figure 4.

Nia et al. [75] also used low-pressure plasma to treat NWPP fabrics prior to grafting with acrylic acid and subsequent coating. Plasma was sustained in oxygen by a capacitively coupled RF discharge operating at the frequency of 50 kHz and RF power of 100 W. The plasma treatment time of 30 s was selected. The WCA for untreated samples was 133°, similar to that shown in Figure 1b. Unlike most other authors who probed the plasma-treated NWPP, Nia et al. [75] did not observe a significant change in the WCA. Furthermore, grafting with acrylic acid also did not change the WCA. The combination, i.e., plasma pre-treatment followed by grafting of acidic acid, enabled an immeasurably low WCA.

Tobias et al. [80] studied the degradation of NWPP fabrics used in surgical masks. They used a capacitively coupled RF discharge operating at 200 W and 40 kHz to sustain a plasma in air at 70 Pa. The intensity of plasma treatment corresponded to the right-hand side of Figure 12, so the authors did not measure the WCAs. Gradual degradation was monitored by weighing the mass during plasma treatment. Selected samples were also probed by XPS. The C1s peak of the untreated samples was narrow and symmetric, indicating no functionalization with polar functional groups. Plasma treatment led to significant oxidation of carbon in the surface film. The authors deconvoluted the C1s peak into C–OH/C–N, O–C=O, and CO_3_ components. An hour of plasma treatment resulted in approximately 15 at.% C–OH/C–N and 7 at.% O–C=O. The carbonate peak became clearly distinguished after treating the NWPP for two hours, and it increased to 25 at.% after 3 h of plasma treatment. Apparently, prolonged plasma treatment favors the formation of carbonate surface functional groups. The results reported by Tobias et al. [80] are marked in Figure 10 in light blue.

Cicogna et al. [79] treated NWPP fabrics used in face mask production with oxygen plasma to promote adhesion of polyphenols. The vacuum discharge chamber was powered with an RF generator at 150 W. The oxygen pressure was 50 Pa, and the treatment time was 20 min. The high-resolution C1s XPS spectra revealed a high concentration of polar functional groups, about 60% C–O, 10% C=O, and 10% O–C=O. Such a composition is not typical for plasma-activated PP but resembles well-activated cellulose [82]. The authors attributed the high concentration of oxygen groups as determined by XPS to the formation of low-molecular-weight fragments. They washed the plasma-treated NWPP and reported removal of the fragments. The results reported by Cicogna et al. [79] are qualitatively consistent with those reported by Tobias et al. [80]: prolonged treatment of NWPP with gaseous plasma results in degradation, and the degradation products have little in common with the original fabric composition.

Recently, Zver et al. [76] used low-pressure oxygen plasma to modify the PP filters of surgical masks. Unlike other authors, he used an inductively coupled RF discharge to sustain gaseous plasma. Such plasma is characterized by excellent energy efficiency because of minimal loss of reactive plasma species in the gas phase (due to the low pressure) and on surfaces (due to the low probability for heterogeneous surface recombination of molecular radicals) [83]. Due to the latter effect, a high density of molecular fragments (atoms in the case of oxygen or nitrogen) is already achieved at low discharge powers [84]. Low-pressure plasma is also a tremendous source of VUV radiation [85]. Not surprisingly, Zver et al. [76] treated NWPP fabrics at the discharge power as low as 10 W. The pressures ranged from 1 to 13 Pa. The initial WCA was 142°, so the wettability shown in Figure 1b applies to these experiments. The immeasurably low WCA was observed in 8 s of plasma treatment at 1 Pa and 3 s at 4 Pa. Treatment at higher pressures did not enable the immeasurably low WCA, probably because of the effect illustrated in Figure 12. The results reported by Zver et al. [76] are marked in Figure 8 and Figure 9 with green-colored symbols.

The team [76] also probed selected samples by XPS. The concentration of functional groups is marked with green-colored symbols in Figure 10. The plasma treatment was found useful for the uniform distribution of an antiviral agent on the fibers within the NWPP fabrics, thereby enabling rapid inactivation of captured viruses (bacteriophage phi6 (DSM 21518)).

### 3.3. General Observations, Discrepancies, and Identification of Scientific Gaps

The results summarized in Figure 8 and Figure 9 are scattered because different teams used different experimental setups. Still, there are some common observations. Considering Figure 8, the vast majority of the WCAs observed after treatment for up to a minute are between 90 and 110°. Obviously, the short treatment time does not allow for the effect illustrated in Figure 2b, i.e., the absorption of a macroscopic water droplet into the fabric. Instead, the short treatment time enables the effect illustrated in Figure 2c, which results from the functionalization of the fibers in the surface layer of the fabrics. However, there is an exemption to this rule: some green-colored symbols in Figure 8 indicate very low (if not immeasurably low) WCA even after a few seconds of plasma treatment. These WCAs were measured at very low pressure (1 or 4 Pa), where the electron temperature is larger due to the lower collision frequency. The higher electron temperature will cause extensive excitation of molecules and atoms to states that are de-excited by radiation in the vacuum ultraviolet part of the spectrum [86]. These photons will not penetrate deeply into solid polymers [87,88], but the NWPP is highly porous, so the photons can reach fibers deep within the fabric. As already mentioned, these photons cause bond scission in polymers, and the resulting dangling bonds are quickly oxidized in low-pressure reactors containing oxygen. The high wettability enables the absorption of liquids into the fabric, as illustrated in Figure 2b. This is desired when fibers throughout the entire fabric are to be grafted with an extremely thin film of a functional material. In the case of surgical masks, the functional material is virucidal.

When considering practical applications, energy efficiency is an important factor. Figure 9 shows wettability as a function of the product of discharge power and treatment time. The green points are well distinguished because the wettability, as illustrated in Figure 2b, is achievable even at the product of a few watt-seconds, making the processing cost-effective. Figure 9 reveals that other plasmas enabled the super-hydrophilic effect only at about 1000 times larger product. Yet another advantage of the low-pressure reactors is scalability. Uniform plasmas could be sustained at the power density (power per volume of glowing plasma) as low as a few kW/m^3^ in volumes of several cubic meters [89]. Here, it should be stressed that energy efficiency depends not only on discharge power and treatment time but also on numerous other parameters, such as the surface area of the treated samples, the materials exposed to plasma, and even the effective pumping speed of the plasma reactor. Unfortunately, few articles disclose these details, so wettability is plotted only as a function of treatment time (Figure 8) and as a function of the product of treatment time and discharge power (Figure 9).

The above discussion appears not to align with the observations reported by De Geyter et al. [52]. The reason is that De Geyter et al. [52] probed pressures down to 300 Pa, where the VUV irradiation of samples is poor, either due to the low electron temperature and thus inadequate excitation of the radiative states, or by gas-phase absorption of the energetic photons, so that the photons emitted from bulk plasma do not reach the polymer sample. Recently, Richmond et al. [85] explained these anomalous results by gas-phase absorption.

The results summarized in Figure 10 are worth discussing as well. The concentration of C–O groups (probably hydroxyl groups) is plotted in Figure 10a versus treatment time. Interestingly, all values are between 10 and 20 at.%, irrespective of the treatment time. The concentration increases significantly only after very long treatment times. A feasible explanation was already provided by Cicogna et al. [79]: formation of LMWFs that have nothing in common with PP. The rapid saturation of the C–O concentration at the surface indicates that a very short treatment time may be sufficient to achieve the maximum possible functionalization with this functional group. However, a significant concentration of these groups may not result in optimal wettability for several reasons [54]. A plot of wettability versus functional group concentration would be useful, but only a couple of the authors reviewed in this article reported both WCA and XPS results.

As discussed above, intensive irradiation of the NWPP with VUV photons would result in a super-hydrophilic surface, as shown in Figure 2. The reason is that the VUV penetration depth in the fabric exceeds that of the reactive plasma species. Another method that also enables super-hydrophilicity of NWPP is treatment with atmospheric-pressure plasma sustained by a nanosecond-pulsed discharge. In this case, the streamers generated by the very fast change in the electric field penetrate the fabrics, as reported by Yang et al. [65]. The effect of such streamers is shown in Figure 7. Such treatments, however, are neither cheap nor scalable.

It is worth noting that not many teams report negative results. An exemption is the article by Wei et al. [74]. They showed that over-treatment induces thermal effects detrimental to wettability. Also, Yang et al. [65] presented SEM micrographs of holes that appeared stochastically on the PP fabrics due to localized fiber melting.

The reviewed articles suggest numerous scientific gaps, which could be summarized as follows. A major scientific challenge is determining the penetration depth. As already discussed, the charged particles cannot penetrate gaps unless the Debye length is much lower than the distance between the neighboring fibers. The neutral radicals (O atoms in particular) can penetrate the gaps regardless of the Debye length, but the concentration decreases with depth because of the loss, which is both by irreversible oxidation of polymer fibers and heterogeneous surface recombination (reaction O + O → O_2_). The flux of VUV photons capable of bond scission will be attenuated, too. The penetration depths of these three species (positive ions, O atoms, and VUV photons) in non-woven polypropylene fabric have yet to be experimentally determined.

The increased wettability is supposed to be a result of sufficient functionalization of the surface fibers with polar functional groups. Interestingly, only a few articles reported both the water contact angles and the concentration of polar functional groups (usually determined by XPS), so a correlation between them remains a scientific challenge.

The aging (hydrophobic recovery) is unavoidable in all cases. Details about the mechanisms are presented in the recent literature [48]. The hydrophobic recovery may be suppressed but not eliminated, so post-treatment (e.g., soaking in appropriate liquids) should be performed as soon as possible after plasma treatment.

Some authors reported damage resulting from inadequate plasma treatment. The damage thresholds have yet to be systematically studied. Preferably, scientists should vary the fluxes of reactive plasma species and report the observed modifications, particularly the melting of the uppermost fibers.

The durability of the coating is another issue that the authors of the reviewed articles have inadequately addressed. Such experiments are time-consuming but necessary for any attempt to use plasma methods on an industrial scale. The scaling-up is a technological challenge. While sustaining uniform plasma in a large volume at low pressure is feasible, atmospheric-pressure plasmas are sustained by streamers, so absolute uniformity is not possible. Still, the high density of streamers ensures sufficient uniformity, taking into account the limitations illustrated in Figure 6 and Figure 7, making roll-to-roll treatment feasible.

Finally, it is necessary to mention the standardization of the wettability tests. The commonly used method is measuring the water contact angle. However, adequate wettability for the absorption of a liquid containing the desired chemical is often achieved when a water droplet is absorbed within a short time, thereby preventing the determination of its contact angle.

## 4. Conclusions

Analysis of results reported by different teams provides guidance for practical applications of plasma techniques for treating NWPP fabrics. The untreated fabrics exhibit a highly hydrophobic character, with water contact angles of 135–145°. If the goal is hydrophilization of the surface film only, many plasmas will do the job. The surface fibers, which come into contact with the plasma, will be quickly functionalized with polar functional groups, resulting in moderate wettability with water contact angles between about 90 and 110°. The hydrophilization of fibers throughout the fabrics, however, requires plasma with well-selected parameters. Based on the literature survey, there are two options to make the fabrics super-hydrophilic without detectable damage to the fabric structure: 1—treatment with atmospheric pressure plasma sustained in a noble gas with an admixture of a reactive gas by a nanosecond pulsed discharge; 2—treatment with low-pressure plasma in the range up to a few Pa. The latter is easily scalable and has low power consumption. Low-pressure plasma appears promising, but industrial suitability still requires validation of throughput, uniformity, aging stability, coating durability, filtration performance, breathability, toxicity, and regulatory compliance.

## Figures and Tables

**Table 1:** Various parameters used by different authors for low-pressure plasma treatments of polypropylene.

Ref.	Gas	Pressure	Electrical Param.	Treatment Time	Reactor Design	PP Sample Type
[55]	CH_4_/O_2_ at 80/20 vol.% and 100 cm^3^/min	~42 Pa	RF plasma at 152 W and 13.56 MHz	5 min plasma; continuous electrospinning at substrate speed 0.325 m/min	Europlasma CD 400 MC low-pressure RF glow-discharge unit; polyvinyl alcohol web deposited in an Elmarco Nanospider NS-Lab (rotating drum electrode, moving textile collector)	100% PP spunbond Vicatex, 30 g/m^2^; antistatic-finished Pegatex S, 16.5 g/m^2^ as ref. collector
[56]	O_2_ plasma; AMPS solution; purged with Ar for 15 min	~15 Pa	NR *	90 s plasma; ~5–40 min UV irradiation; 30 min maximum graft density	DT-03 plasma apparatus followed by a high-intensity UV-lamp grafting setup	NWPP membrane with an avg. pore diameter of 0.22 μm, Beijing JDKR
[57]	O_2_ plasma; monomer solution purged with Ar for 15 min	~15 Pa	Plasma at 120 W	90 s was selected (plasma time varied); ~5–60 min UV exposure (~30 min used in several later experiments)	DT-03 plasma apparatus; UV grafting conducted with monomer solution in a transparent polystyrene round-bottom tube beneath a high-intensity UV lamp	Circular NWPP membrane, 3.6 cm diameter, avg. pore diameter 0.22 μm, Beijing JDKR
[58]	O_2_; acrylic-acid solution purged with N_2_ for 20 min	~4 Pa base; 15 Pa O_2_ working	Plasma at 30 W	3 min plasma; 4 h at 80 °C acrylic-acid grafting; EDC activation and heparin reactions 24 h per step	HD-1B low-temperature plasma with a rotary-vane pump; subsequent grafting in glass vessels and a temp. regulated water bath	NWPP fabric, 148 g/m^2^, Shanghai Shilong Hi-tech; 10 cm × 10 cm
[59]	Aqueous monomer system purged by N_2_ bubbling for 10 min	NR	^60^Co γ radiation (dose up to ~55 kGy); grafting plateaued at ~13 kGy; dose rate 0.11–1.67 kGy/h	Dependent on dose and dose rate; one main concentration series used 10 kGy over 17 h	NWPP and aqueous N-vinyl-2-pyrrolidone placed in sealed glass tubes and irradiated with a ^60^Co γ-ray source	NWPP fabric, 130 g/m^2^, 10 cm × 10 cm, Shanghai Shilong Hi-tech
[60]	O_2_ plasma; samples exposed to air after treatment	~15 Pa	NR	90 s plasma; 0–15 min UV time (8 min selected); 1 h at 4 °C EDC/NHS activation	DT-03 plasma apparatus; for photografting, membranes infiltrated with 200 μL of 20% *v*/*v* acrylic acid and placed between two quartz plates beneath a homemade high-intensity UV lamp	Circular NWPP membrane, 3.6 cm diameter, avg. pore diameter 0.22 μm, Beijing JDKR
[61]	O_2_ plasma	4–6 Pa; dip coating and drying at 25 mbar	RF plasma at 50 W	5 min plasma; 30 min low-vacuum immersion; 145 °C for 5 s final thermal fixation	Dionex 2000 RF plasma reactor with cylindrical quartz chamber, 10-inch internal diameter × 20-inch depth; the 2-inch cartridge was mounted centrally on a specially designed aluminum hanger	2-inch MBPP depth-cartridge filter, Borealis Borow HL504FB; fiber diameters approximately 0.2–30 μm
[62]	Air plasma at flow 0.03–0.04 g/s for activation; Ar + propane-butane at flow 0.01–0.04 g/s for fixation	10–30 Pa	Air-plasma activation at 1.2–2.4 kW; Ar/hydrocarbon fixation at 0.7–2.2 kW	60–600 s each plasma stage (treatment and fixation); 10–20 min at 20–24 °C nanoparticle impregnation	Experimental vacuum RF plasma installation developed at KNRTU	Spunbond NWPP 50 g/m^2^, JSC Polimatiz
[63]	Ar 180 sccm + N_2_ 20 sccm	6.7 × 10^−4^ Pa base; 0.23 Pa working	450 W, 450 V, 1 A, 124 mA/m^2^; negative square DC pulses at 60 kHz and 50% duty cycle	20 s at room temperature; 100 mm/min roll speed	Modified DC-pulsed magnetron sputtering integrated with roll-to-roll transport; graphite target 0.416 m × 0.298 m with a target-to-fabric distance of 6 cm	NWPP fabric

* NR = not reported.

## Data Availability

No new data were created or analyzed in this study. Data sharing is not applicable to this article.

## Abbreviations

The following abbreviations are used in this manuscript:

PP	Polypropylene
NWPP	Non-woven polypropylene
WCA	Water contact angle
DC	Direct current
MW	Microwave
APPJ	Atmospheric pressure plasma jet
MBPP	Melt-blown polypropylene
XPS	X-ray photoelectron spectroscopy
SIMS	Secondary ion mass spectrometry
FTIR	Fourier transform infrared spectroscopy
AFM	Atomic force microscopy
SEM	Scanning electron microscopy
LMWF	Low-molecular-weight fragment
DBD	Dielectric barrier discharge
VUV	Vacuum UV
